# Solid Organ Transplantation and Body Contouring Surgery: A Case Series and Single-Center Experience

**DOI:** 10.3390/jcm15176549

**Published:** 2026-08-25

**Authors:** Philipp Tratnig-Frankl, Andrea Wiland, Christian Freystätter, Eva Placheta-Györi, Christine Radtke

**Affiliations:** Department of Plastic, Reconstructive and Aesthetic Surgery, Vienna General Hospital, Medical University of Vienna, 1090 Vienna, Austria

**Keywords:** body contouring surgery, single organ transplantation, bariatric surgery, postoperative care, plastic surgery

## Abstract

**Background:** In recent years, the number of patients seeking body contouring surgeries after a single organ transplantation has increased due to better postoperative care, better immunosuppressive regimens and the advancement of laparoscopic bariatric surgical techniques. In these patients, body contouring procedures can improve function, aesthetics, and overall quality of life and are offered to a greater number of patients including cohorts with significant medical comorbidities such as immunosuppression after solid organ transplantation. Therefore, in this case series, we explored the topic of body contouring surgery in patients who underwent solid organ transplantation. **Methods:** In this retrospective study, we included patients who underwent a single organ transplantation prior to a body contouring surgery between 2010 and 2021. Inclusion criteria were age over 18 years, a body contouring surgery at our center, a single organ transplantation before the body contouring procedure and a postoperative follow up of four weeks or more. **Results:** A total of 422 patients underwent body contouring procedures in our center. After excluding non-eligible patients, four patients were recruited. Overall, four complications in three patients occurred, ranging from Grade I to II according to the Dindo and Clavien classification for surgical complications. **Conclusions:** Body contouring surgery seems to be safe in patients with single organ transplantation. However, careful patient selection in a multidisciplinary team is required to ensure patients’ safety and favorable postoperative outcomes in patients with extended operative indications and significant comorbidities such as solid organ transplantation and immunosuppression. Complex patients should be treated in close collaboration with transplant surgery and internal medicine at a tertiary center.

## 1. Introduction

The relationship between obesity and surgical complications is multifactorial and increasingly relevant given the rising prevalence of obesity among surgical patients with increased risk of certain surgical complications, most notably surgical site infections, venous thromboembolism, and renal complications [1,2,3,4,5]. On the other hand, the numbers of liver and kidney transplantation have increased, with a total of 1659 liver transplantations and 28,142 kidney transplantations in the United States in 2023 [6,7]. Common indications for liver transplantation are non-alcoholic fatty liver disease (NAFLD), non-alcoholic steatohepatitis (NASH), hepatocellular carcinoma (HCC) and alcohol-related liver disease (ALD) [8,9,10,11,12]. On the other hand, the most common causes of chronic kidney disease (CKD) and end-stage kidney disease (ESKD) are diabetes and hypertension, which account for the majority of patients requiring dialysis or kidney transplantation [13,14,15,16].

Especially obese patients, defined by a body mass index (BMI) ≥ 30 kg/m^2^, have higher risks of developing metabolic disorders and are therefore prone to organ dysfunctions [17,18,19]. In order to minimize the complications of obesity and its comorbidities, bariatric surgery (BS) has become a validated surgical option for treating obesity in patients suffering from end-stage renal disease, decreasing the percentage of graft failure in organ recipients and reducing the burden of comorbidities [20,21,22]. Body contouring surgeries are intended to excise loose or excessive skin, which is often a result after bariatric surgical procedures [23].

It has to be taken into consideration that from a non-surgical point of view, body contouring procedures (e.g., abdominoplasty) have been associated with significant improvements in psychosocial outcomes and body image, as quantified by validated instruments such as the BODY-Q [24,25]. However, post-bariatric patients were found to have higher rates of postoperative complications after body contouring procedures, including surgical side infections, seroma formation and prolonged wound healing in up to 42.9% of patients, compared to 12.6% in a non-bariatric cohort [26]. In the past, patients that were primarily not considered for single organ transplantation (SOT) because of their comorbidities are now given further surgical options in order to become possible candidates for SOT [20,27,28]. For surgical planning, it has to be considered that each abdominal surgery impairs the blood supply of the abdominal wall as scarring interrupts the zones of perfusion, which may deteriorate wound healing in subsequent surgeries [29,30].

With increasing survival rates, organ recipients are seeking body contouring surgeries as well, which can be challenging due to their history of transplantation [31].

The aim of this study was the assessment of perioperative complications in patients with body contouring surgeries after single organ transplantation.

## 2. Materials and Methods

Patients treated with surgery for body contouring at a tertiary academic plastic surgery department between January 2010 and September 2021 were included in this retrospective, monocentric study. In total, 592 body contouring surgeries were performed in 422 patients. Of these patients, four (*n* = 4) had a body contouring procedure after the single organ transplantation. Patients with an incomplete medical history were excluded from this study.

### 2.1. Patients

Patients’ demographics, comorbidities, transplanted organ, immunosuppressive maintenance regimens, length of hospital stay, and postoperative course were assessed. The classification proposed by Dindo et al. for surgical complications was used to assess patient outcomes [32]. All patients were carefully evaluated preoperatively in a multidisciplinary setting involving internal medicine specialists and transplant surgeons. This assessment included a thorough review of graft function and viability. At the time of the BCP, patient #3 was totally weaned off his immunosuppressive medication, patient #1 and patient #2 had a monotherapy (either FK-506 or Cyclosporine-A), and one (patient #4) continued being on triple immunosuppression.

### 2.2. Statistical Analysis

Descriptive statistical analysis was performed for all assessed data, such as patient demographics, bariatric procedures, weight loss, BCP, SOT and postoperative complications. Microsoft Excel^TM^ (version 16.27, 2019 Microsoft Corporation, Redmond, WA, USA) was used for statistical analysis.

## 3. Results

A total of four patients (*n* = 4) were included (Table 1): one female and three male patients with a mean age of 46 years (range: 28 to 56 years). Apart from having a SOT, all male patients underwent a gastric sleeve procedure before they were referred to our clinic for BCP. The female patient did not have any bariatric surgery. The mean preoperative weight was 73.25 kg (range: 61 to 83 kg), while the preoperative body mass index (BMI) was 25.2 (range: 22.3 to 28.4).

Transplanted organs included two liver, one heart and a kidney transplantation. Most of our patients had a minimum of three comorbidities, including arterial hypertension or diabetes. The time period between the SOT and their BCP was on average 6.6 years (range: 3.1 to 9.0 years).

### 3.1. Body Contouring Procedures

**Figure 1:**
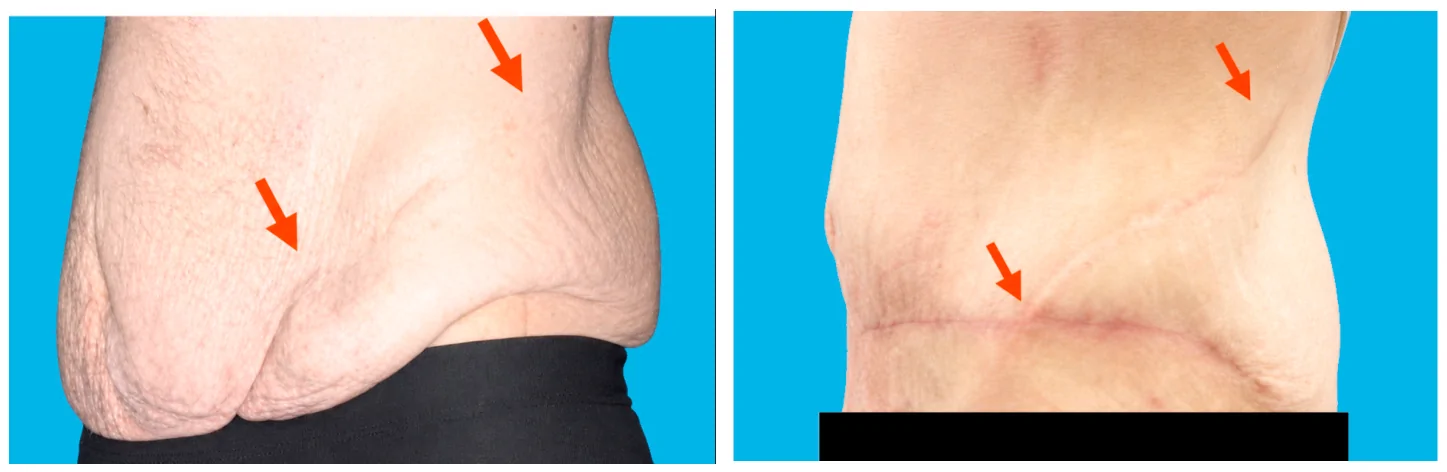
A 49-year-old male patient with a history of kidney transplantation and subsequent gastric sleeve gastrectomy resulting in 30 kg weight loss, who underwent abdominoplasty. Preoperative view (**left**) and postoperative result at 12 weeks (**right**). Red arrows indicate the scar from a kidney transplantation performed 37 months prior to body contouring surgery.

The male patients had an abdominal dermolipectomy, due to concerns about further harming the blood supply of the abdominal wall after liver or kidney transplantation (Figure 1). The female patient underwent a bilateral thigh lift (Table 2). The intraoperative resection weights were 1200, 1084 and 380 g in the male patients and a total of 575 g in the female patient undergoing the bilateral thigh lift. Intraoperatively, no complications occurred, and the median postoperative hospital length of stay was 12.5 days (7 to 20 days).

### 3.2. Complications in Patients Undergoing Body Contouring Surgery After Single Organ Transplantation

Postoperatively, one patient had an uneventful course and was discharged after seven days. The next patient developed small dermal purpura postoperatively, which was diagnosed as thrombophilic vasculitis. This was most likely attributable to a preoperatively unrecognized APC resistance. As the symptoms were self-limiting, no further therapeutic intervention was necessary (Grade I).

In the same patient, an abscess formation was found in the lower pelvis, which was then treated with an antibiotic course (Grade II). Both complications were not related to the initial surgical procedure. Another patient caught a respiratory infection, with leukocytosis of 12.3 G/L and an elevated C-reactive protein level of 13.1 mg/dL, so an intravenous antibiotic course was started and continued orally after discharge. This was graded as Grade I.

The next patient had a prolonged postoperative seroma, with the drains kept until the 11th postoperative day, which was classified as a Grade I complication. No abnormal elevations were observed in the patient’s laboratory findings.

None of these patients suffered other complications within the first four weeks of the postoperative period (Table 2).

## 4. Discussion

It can be assumed that the number of patients undergoing transplantation and body contouring surgery afterwards is going to increase. Both bariatric surgery and body contouring surgery seem to improve the quality of life postoperatively in patients dealing with excess skin [24,25,33].

So far, there is little data on patients with single organ transplantation undergoing body contouring procedures and whether they are more likely to have postoperative complications [31,34]. In a recent work, published by Iannelli et al., the outcome of a liver transplantation was not affected by previous BS [35]. However, patients after BS undergoing BCP do have reported complication rates up to 37% [36,37]. This includes minor complications like seroma or wound dehiscence, which partially correlates with our findings.

A meta-analysis performed by Hasanbegovic et al. showed that in post-bariatric patients undergoing BCP, the risk of a complication increases by 60% compared to patients who lost weight due to other reasons [38]. If transplantation is required, obesity can be associated with organ damage and therefore a progression of its failure, while an increased BMI prolongs the patient’s time listed for transplantation [39]. Dziodzio et al. state that bariatric surgery is a valuable surgical option for obese patients facing kidney transplantation or even for organ recipients [20], especially because some patients after SOT gain 30% of their body weight within three years after transplantation [40]. For that reason, BS should be considered to reduce body weight in order to prevent transplant failure [22].

On the other hand, standard surgical incisions for SOT can compromise the blood supply of the abdominal wall and make subsequent procedures (e.g., an abdominoplasty) technically more demanding [41,42,43]. When considering a transplant patient for BCP, a key factor possibly impeding the operative results is an ongoing immunosuppressive therapy. Although these regimens vary between transplant centers, most often calcineurin inhibitors, mycophenolate mofetil and corticosteroids are used [44].

In the current literature, wound healing complications after kidney transplantation are reported in up to 27% of the patients [45,46,47]. Corticosteroids are especially associated with an impairment of wound healing [48]. We were not able to correlate these findings in our study due to the low patient numbers.

Another recent publication compared the quality of life in post-bariatric patients who underwent body contouring surgery (e.g., abdominoplasty or panniculectomy) to post-bariatric patients who did not have BCP. The authors were able to show a significant improvement in the quality of life in these patients, measured with the BODY-Q score [25]. Similar results with a two-year follow-up period were shown by Song et al., where the authors were able to demonstrate that bariatric surgery and body contouring surgery can enhance the quality of life and body image in these patients [49].

Among our patients, one patient had undergone kidney transplantation—which is statistically the most commonly performed single organ transplantation [50]. Before undergoing BCP, some patients were already weaned off the standard triple immunosuppression, with one patient even being weaned off his immunosuppressive therapy completely. As mentioned above, most of our patients underwent dermolipectomy to not jeopardize the blood supply of the abdominal wall after a SOT had already been performed.

Although we included patients from the past ten years, this single-center study comprises a small case series; yet, complication rates seem to correlate with other publications already available. Study limitations include the low patient number and the retrospective study design. While no major complications were observed in our cohort, this limited number precludes a robust and generalizable assessment of the safety profile of body contouring procedures in transplant patients.

Larger, prospective, and ideally multicenter studies are needed to more comprehensively characterize the safety profile, identify potential risk factors for complications, and establish more definitive conclusions regarding the overall risk–benefit ratio of this approach. Future studies should therefore incorporate extended follow-up periods together with patient-reported outcome measures, as these would provide a more comprehensive understanding of how body contouring surgery affects quality of life and functional status in this specific patient population.

Future studies should also address the effect of the pharmacological treatment of obesity, with Obesity Management Medication, to study its effect on wound healing in patients who underwent single organ transplantation [51,52].

## 5. Conclusions

Body contouring surgery appears to be a safe procedure in patients who have undergone single organ transplantation, with complication rates comparable to those reported in the general post-bariatric population. As the number of transplant recipients seeking body contouring procedures continues to rise, these patients should not be categorically excluded from surgical candidacy. However, due to the complexity of this cohort, management should be conducted at tertiary centers with close interdisciplinary collaboration between plastic, transplant, and internal medicine specialists.

## Figures and Tables

**Table 1:** Patient demographics. SOT (single organ transplantation), IS (immunosuppressive regiment), BS (bariatric surgery), and BMI (body mass index).

Patient	Age	Gender	SOT	IS Preoperatively	BS	Comorbidities	Smoking	BMI
#1	56 y	Male	Liver	FK-506	Gastric Sleeve	≤3	Unknown	26.3
#2	51 y	Female	Liver	Cyclosporine	None	>3	No	23.8
#3	28 y	Male	Heart	None	Gastric Sleeve	>3	No	28.4
#4	49 y	Male	Kidney	FK-506, MMF, Steroids	Gastric Sleeve	>3	No	22.3

**Table 2:** Body contouring procedure (BCP) and complications, classified according to Clavien and Dindo, and length of hospital stay (LOS).

Patient	BCP	Resection Weight (g)	Complications	Classification	LOS (d)
#1	Abdominal dermolipectomy	1084	None	N/A	7
#2	Bilateral thigh lift	575	Thrombophilic vasculitis, Pelvic abscess formation	Grade I Grade II	20
#3	Abdominal dermolipectomy	380	Respiratory infect	Grade II	9
#4	Abdominoplasty	1200	Prolonged postoperative seroma	Grade I	14

## Data Availability

Data supporting the conclusions of this article will be made available by the authors on reasonable request.

## Abbreviations

The following abbreviations are used in this manuscript:

BCP	Body contouring procedure
SOT	Single organ transplantation
BS	Bariatric surgery
